# Case Report: Hyponatremia Secondary to Desmopressin Administration Prior to Percutaneous Kidney Biopsy: A Case-Based Review

**DOI:** 10.3389/fmed.2021.696904

**Published:** 2021-06-21

**Authors:** Alexandra Vornicu, Bogdan Obrişcă, Bogdan Cotruta, Adriana Octaviana Dulămea, Nicu Caceaune, Gener Ismail

**Affiliations:** ^1^Department of Nephrology, Fundeni Clinical Institute, Bucharest, Romania; ^2^“Carol Davila” University of Medicine and Pharmacy, Bucharest, Romania; ^3^Department of Gastroenterology and Hepatology, Fundeni Clinical Institute, Bucharest, Romania; ^4^Department of Neurology, Fundeni Clinical Institute, Bucharest, Romania; ^5^Department of Internal Medicine, Fundeni Clinical Institute, Bucharest, Romania

**Keywords:** desmopressin, hyponatremia, kidney biopsy, gastrointestinal bleeding, cerebral edema, case report

## Abstract

Bleeding remains the most clinically relevant complication of kidney biopsy and several prophylactic approaches were proposed, including desmopressin administration. We present the case of a 60-year-old man with a history of liver transplantation, admitted for the evaluation of a renal dysfunction. As part of our department protocol, desmopressin 60 μg was administered orally, 2 h before the percutaneous kidney biopsy. The patient developed acute, severe, symptomatic hyponatremia (i.e., headache and recurrent vomiting), followed by a life-threatening upper gastrointestinal bleeding due to a Mallory-Weiss syndrome. Although it is often used as bleeding prophylaxis prior to kidney biopsy, data regarding the efficacy and safety of desmopressin in this setting are inconsistent. Accordingly, we performed a thorough literature review of the use of desmopressin as bleeding prophylaxis prior to kidney biopsy, focusing on the incidence of hyponatremia. The reported incidence of hyponatremia (<130 mmol/l) was 7–11%, probably because serum sodium was monitored in few studies. Nevertheless, hyponatremia was rarely symptomatic but, in some cases, like the one presented here, its complications could be severe. Pre-biopsy low serum sodium and estimated glomerular filtration rate as well as high spot urine sodium and non-restricted fluid intake were reported to be associated with hyponatremia incidence. However, the current evidence cannot clearly establish which patients benefit the most from desmopressin use with respect to bleeding complications. We propose that when desmopressin is used for bleeding prophylaxis prior to kidney biopsy, measurements of serum sodium levels, before and every 6 h after, should complement ultrasound and hemoglobin as part of the patient post-procedural monitoring. Also, water intake should be restricted in the day of biopsy. However, this proposed approach should be adequately evaluated in a clinical trial.

## Introduction

The kidney biopsy remains the gold standard for diagnosis, guiding the treatment and prognosis in patients with glomerular and tubulointerstitial disorders (1). Since its initial description in 1951 by Iversen and Brun (2), the kidney biopsy technique has significantly improved in order to obtain an adequate tissue sample and to minimize the risk of complications (1, 3–5). Of these, bleeding remains the most clinically relevant complication (1, 3–5).

Post-biopsy bleeding rates vary significantly across different studies, in part due to heterogeneity in definitions of bleeding complications or in diagnostic algorithm, while several prophylactic approaches were been proposed (3, 4, 6). Of these, desmopressin, a synthetic vasopressin receptor agonist, was shown to reduce bleeding time in uremic patients by transiently increasing von Willebrand factor and factor VIII blood levels, and was recommended for bleeding prophylaxis prior to kidney biopsy (7–9). However, desmopressin can lead to iatrogenic acute, sometimes severe, hyponatremia if appropriate fluid restriction is not achieved (10, 11), with subsequent brain edema and neurologic symptoms, varying from mild nausea and vomiting to lethargy, headaches and confusion, eventually followed by severe neurologic sequalae (12).

We describe here the case of a liver transplant recipient who underwent a kidney biopsy for clinical concern of calcineurin inhibitor nephrotoxicity. Following desmopressin administration, the patient developed acute, severe, symptomatic hyponatremia that was complicated by a Mallory-Weiss syndrome with massive gastro-intestinal bleeding. Accordingly, we performed a thorough literature review of desmopressin use as bleedings prophylaxis after kidney biopsy focusing on the risk of hyponatremia occurence.

## Case Description

A 60-year-old man was admitted in our nephrology department for the evaluation of a renal dysfunction. His past medical history included a hepatitis B virus and hepatitis D virus chronic co-infection. His liver disease progressed to a decompensated cirrhosis, he had five episodes of variceal hemorrhages resolved by endoscopic variceal ligation, and underwent a whole liver orthotopic transplantation 1 year prior to the admission.

Although the estimated glomerular filtration rate (eGFR) decreased immediately after liver transplantation, the patient had a stable serum creatinine (1.8 mg/dl) in the last year. Before liver transplantation, the renal function was normal, and no electrolyte disturbances were recorded. The patient did not have any prior episodes of hyponatremia. Additionally, post-transplant follow-up endoscopies did not reveal any esophageal varices and the patient did not have, at the time of admission, any clinical concerns for a gastro-intestinal hemorrhage.

His current medication included the maintenance immunosuppressive therapy, tacrolimus (with trough levels between 5 and 10 ng/ml) and mycophenolate mofetil (1 g per day), and entecavir.

At admission, the patient was otherwise well, he didn't have any edema, his blood pressure was 130/70 mmHg and heart rate of 65/min. The abdominal palpation did not reveal any hepatomegaly, splenomegaly or signs of ascites. His urine output was 2,500 ml/day. The rest of the clinical exam was unremarkable.

The initial laboratory evaluation showed an increased serum creatinine (1.7 mg/dl) and urea (56 mg/dl), and mild hyperkalemia (5.7 mmol/l) (Table 1). The serum sodium was within normal range (139 mmol/l). The complete blood count and the coagulation profile were within normal ranges. Liver tests revealed no cytolysis; there were no signs of viral replication. Urinalysis revealed a bland sediment, without proteinuria.

**Table 1:** Laboratory findings.

**Laboratory test**	**Admission**	**12 h post-renal biopsy**	**36 h post-renal biopsy**	**Discharge**	**Reference ranges**
Serum creatinine (mg/dl)	1.7	1.7	2	1.8	0.5–0.9
Serum urea (mg/dl)	56	60	58	62	10–43
Serum uric acid (mg/dl)	5.45	6.2	5.4	5.4	2.4–5.7
Serum sodium (mmol/l)	139	123	130	139	136–145
Serum potassium (mmol/l)	5.7	5.6	5.2	5.5	3.5–5.1
Serum glucose (mg/dl)	85	87	82	85	60–99
Serum osmolality (mosm/kg H_2_O)	303	268	285	301	280–300
Urine osmolality (mosm/kg H_2_O)	250	600	455	263	50–1,200
Urine sodium (mmol/L)	30	45	40	32	54–150
Hemoglobin (g/dl)	13	9	8	9	12.3–15.3
Platelet count (cell*1,000/mmc)	245	236	250	241	150–450
TSH (μUI/ml)	2.3	–	–	–	0.27–4.2
Serum cortisol (nmol/l)	250	–	–	–	172–497

A percutaneous ultrasound-guided kidney biopsy was performed. As part of our department protocol, desmopressin 60 μg was administered orally, 2 h before the procedure. The post-procedure ultrasound examination after 5 min, 1 and 24 h did not reveal any bleeding of renal origin.

**Figure 1:**
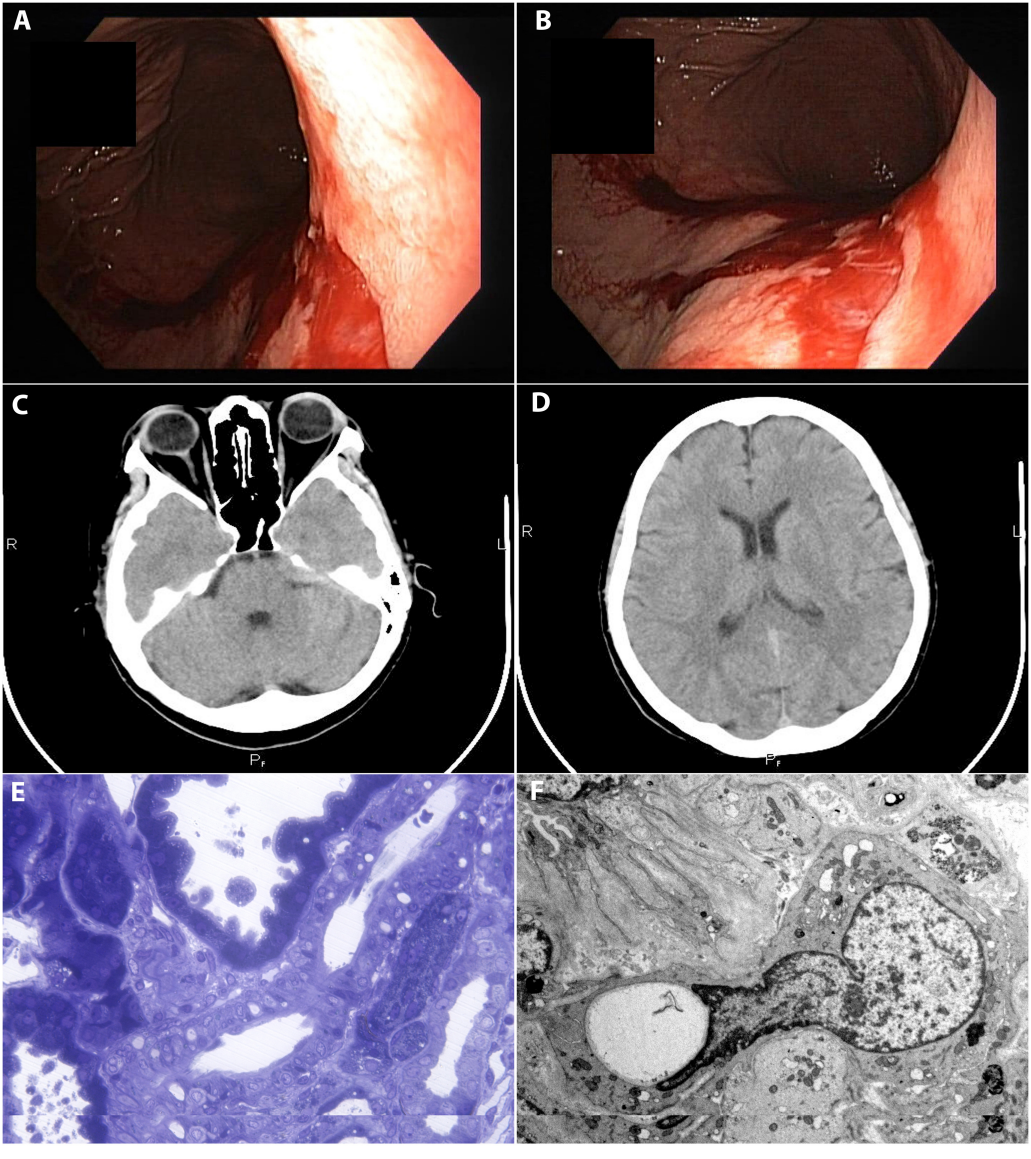
**(A,B)** Eroded area at the posterior face of the cardia below the Z line suggestive for Mallory-Weiss syndrome. **(C,D)** Diffuse cerebral and cerebellar edema associated with acute hyponatremia. **(E,F)** Kidney biopsy. Calcineurin inhibitor nephrotoxicity. **(E)** Light microscopy of a section stained with toluidine blue staining. **(F)** Electron Microscopy.

In the first 12 h of monitoring, the patient was otherwise well, without any clinical symptoms, did not receive any iv fluids nor ingest an excess of free water. However, 12 h post-biopsy, the patient developed headache, nausea and two episodes of vomiting, followed by a massive upper gastrointestinal bleeding. The sodium concentration acutely decreased to 123 mmol/l, while hemoglobin level dropped by 4 g/dl. He was admitted in the intensive care unit and an urgent endoscopy was done that revealed characteristic findings for Mallory-Weiss syndrome: an eroded area on the posterior face of the cardia below the Z line, without any signs of esophageal varices (Figures 1A,B). The neurological examination and a CT scan confirmed the brain edema (Figures 1C,D).

After 24 h of apparent stabilization, he developed a second episode of upper gastrointestinal bleeding, with another drop of 1 g/dl in hemoglobin. A second endoscopy showed small areas of ulcerations along the right border of the gastroesophageal junction without active bleeding.

**Figure 2:**
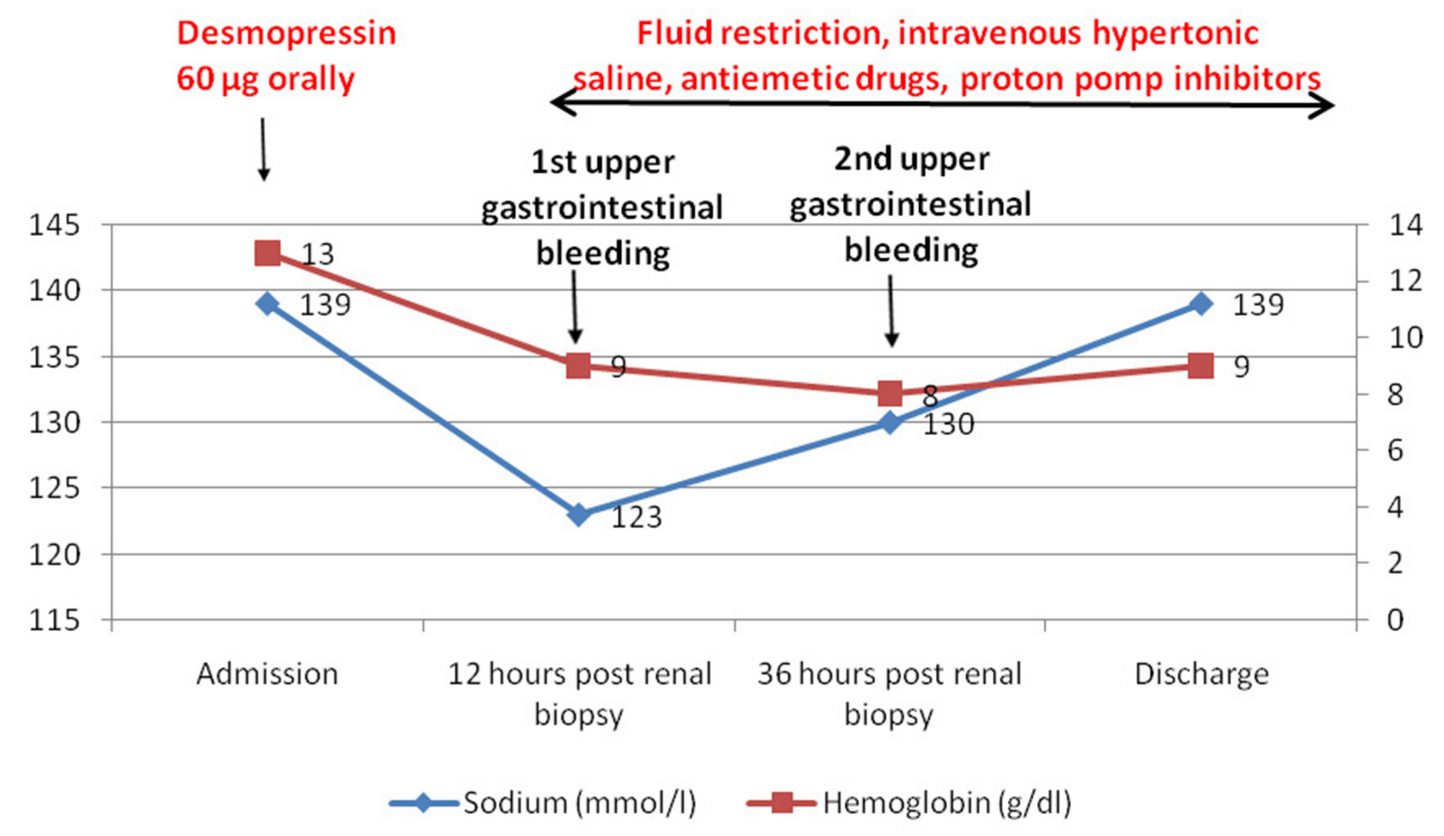
Timeline of the episode of care showing the evolution of serum sodium and hemoglobin levels.

During admission, hyponatremia was gradually corrected by fluid restriction for 48 h (<1 L/day) and intravenous 3% hypertonic saline solution (Figure 2). Hypertonic saline solution was given at a dose of 1 mL/kg/h with serial laboratory measurements every 2 h (for a total of 300 mEq of sodium administered). Serum sodium increased by 6 mEq/l by 12 h and by 7 mEq/l by 24 h, while the neurological symptoms completely resolved (Table 1). The gastrointestinal bleeding was conservatively managed with antiemetic drugs and proton pomp inhibitors.

The kidney biopsy was consistent with calcineurin inhibitor nephrotoxicity (Figures 1E,F).

## Discussion

We described, to our knowledge, the first case of Mallory-Weiss syndrome in the setting of acute hyponatremia induced by desmopressin administration prior to a percutaneous kidney biopsy. We propose that when desmopressin is used for bleeding prophylaxis after kidney biopsy, especially in those at high risk of hyponatremia (low baseline serum sodium, low eGFR), serum sodium levels should be assessed before and every 6 hours thereafter, complementary to ultrasound and hemoglobin, as part of the post-procedure monitoring, and hypotonic fluid intake should be limited.

Given the inconsistency of data regarding the safety, particularly hyponatremia incidence, of desmopressin given for bleeding prophylaxis after percutaneous kidney biopsy, we performed a thorough literature review of published reports (Table 2).

**Table 2:** Studies reporting hyponatremia and bleeding after prophylactic desmopressin (DDAVP) administration prior to kidney biopsy.

**References**	**Type of study**	**Biopsies**		**DDAVP prophylaxis**		**Hyponatremia**	**Bleeding**
		**Total (n)**	**DDAVP (n)**	**Criteria for administration**	**Route and dose**		
Manno et al. (13)	Double-blind RCT	162	80	Randomization	0.3 μg/kg, SC	No symptomatic hyponatremia	DDAVP significantly decreased the relative risk of bleeding with 45% and hematoma size in the 36 patients with normal kidney function (eGFR > 90 mml/min) who experienced bleeding
Anandagoda et al. (14)	Case report	2	2	NR	12 μg, IV	Profound hyponatremia (107 mmol/l and 124 mmol/l) with neurological sequelae	No macroscopic hematuria
Tsai et al. (15)	Retrospective	269	269	Routine administration	4 units (infusion in all patients)	No symptomatic hyponatremia	Without control group The total complication rate was of 5.2%
Athavale et al. (16)	Retrospective	269	100	Decision left to nephrologist	0.3 μg/kg, IV	No symptomatic hyponatremia	DDAVP decreased bleeding risk (OR 2.11) in patients with sCr ≥ 1.8 mg/dL but increased bleeding risk (post-biopsy drop in hemoglobin) in those with sCr < 1.8 mg/dl (OR 9.72)
Lim et al. (11)	Retrospective	436	226	Physician dependent, but suggested if serum urea > 15 mmol/L, sCr > 200 μmol/L or eGFR <30 ml/min	Median dose 0.20 (0.17, 0.24) μg/kg, IV	DDAVP was associated with severe hyponatremia (10.7 vs. 3.0%, *p* 0.002)	No significant differences in the occurrence of minor bleeding (13.7 vs. 12.9%, *p* 0.79) and major bleeding (7.5 vs. 4.3%, *p* = 0.15)
Ho et al. (17)	Retrospective	195	98	Physician dependent, but suggested if serum urea > 15 mmol/L, sCr > 200 μmol/L or eGFR <30 ml/min	0.3 μg/kg, IV	DDAVP increased the risk of hyponatremia with 26% (*p* = 0.03) if fluid intake was not <1 L in the day of biopsy.7 cases of severe hyponatremia (7%) in the DDAVP group but none in the non-DDAVP group	No significant differences in incidence of overall bleeding (8.2 vs. 8.2%, *p* = 1.00), minor bleeding (6.1 vs. 8.2%, *p* = 0.59) and major bleeding (5.1 vs. 2.1%, *p* 0.45) in those with DDAVP as compared to those without
Leclerc et al. (18)	Retrospective	413	328	Decision left to nephrologist	0.3 μg/kg, IV	DDAVP was associated with acute hyponatremia (10 vs. 4%; *p* 0.08); no severe symptomatic hyponatremia	Similar likelihood (OR 0.39) of symptomatic hematomas and a lower need for urgent angiography in patients at high (eGFR 28 ml/min) and low (eGFR 45 ml/min) risk of bleeding
Rao et al. (19)	Prospective and retrospective	194	89	sCr > 132.4 μmol/L and/or eGFR <60 mL/min	150 μg, intranasally	Natremia decreased in 94% of patients (median decrease 4.4 mmol/l).In 9%, natremia was <130 mmol/l, but hyponatremia was asymptomatic	Significant lower incidence of overall bleeding (15.7 vs. 31.4%, *p* = 0.02) and self-limited gross hematuria (7.8 vs. 13.4%, *p* = 0.03) in DDAVP group. Major complications (need of transfusions, angiography) were not significantly different

*BT, bleeding time; DDAVP, desmopressin; eGFR, estimated glomerular filtration rate; n, number; OR, odds ratio; IV, intravenously; SC, subcutaneously; sCr, serum creatinine*.

Bleeding remains the most clinically relevant complication of kidney biopsy (1). However, there is a large variation in incidence of the bleeding complications requiring transfusions across studies, ranging from 0.9 to 9%, explained by the different definitions of bleeding complications, by the heterogeneity of protocols and patients, and by the retrospective nature of most studies (3, 4, 6).

Desmopressin is a synthetic selective endothelial vasopressin-2 receptors agonist which rapidly and transiently (90–120 min) increase plasma levels of factor von Willebrand factor and factor VIII (7). It enhances platelet adhesion and transiently releases tissue plasminogen activator into plasma (7). Accordingly, it was recommended for bleeding prophylaxis prior to various procedures, including kidney biopsy (9). However, desmopressin also stimulates vasopressin-2 receptors in the collecting duct, increasing free water reabsorption, by inserting aquaporins into the nephrocytes membranes (10, 11).

Desmopressin pharmacokinetics depends on the route of administration. While irrespective of route, the peak plasma concentration is attained after 1 h, the area under the curve of plasma concentrations is the highest when administered subcutaneously, followed by the intravenous, intranasal and oral routes (20, 21). As kidney is a site of desmopressin degradation (20), in patients with a low eGFR, its actions are prolonged up to 8 h (8).

Desmopressin was shown to reduce bleeding time in uremic patients (8) and was recommended for bleeding prophylaxis prior to kidney biopsy (9). Although it is often prescribed, there are insufficient data to conclude on its utility and safety in this setting (11).

Initially, desmopressin was indicated prior to renal biopsy only in patients suspected or previously diagnosed with thrombophilia, and in those with a prolonged bleeding time (13, 22–26). As the relation between bleeding time and bleeding complications after renal biopsy was not uniformly proved, the need of assessing bleeding time was thereafter questioned (23, 24, 27). Later, clinicians started to use desmopressin prophylactically, in patients presumed to have a high bleeding risk because of reduced kidney function (eGFR < 30–45 ml/min/1.73 m^2^ or serum creatinine higher than 1.7–2.8 mg/dl) (11, 17, 19, 28–31).

Only one randomized controlled study evaluated the efficacy of desmopressin administration (0.3 μg/kg, subcutaneously) prior to kidney biopsy and concluded that it has beneficial effects in terms of decreasing by 45% the relative bleeding risk and hematoma size, but in patients with preserved kidney function (eGFR ≥60 ml/min), thus at low risk of bleeding (13).

Currently, data in patients with eGFR <60 ml/min/1.73 m^2^ are derived from observational, retrospective studies with conflicting results (4, 11, 15–19, 28, 31). Some studies suggested a beneficial effect of desmopressin use in patients with reduced kidney function (15, 16, 28, 31). Peters et al. conducted a multicenter study to evaluate the efficacy of desmopressin (0.3 μg/kg subcutaneously) for reducing the bleeding risk after kidney biopsy, in patients with serum creatinine >1.7 mg/dl. The frequency of bleeding complications was evaluated in one group of patients followed in a center who received desmopressin, and compared to a cohort of patients followed in five other centers who did not receive desmopressin. Desmopressin reduced overall bleeding complications (3.5 vs. 8.4%) (28). Similarly, in a recently study by Rao et al., administration of desmopressin prior to renal biopsy was evaluated in patients with serum creatinine > 1.5 mg/dl and/or eGFR <60 ml/min/1.73 m^2^. Desmopressin administration reduced overall bleeding events (15.7 vs. 31.4%, *p* = 0.02) and self-limited gross hematuria (7.8 vs. 13.4%, *p* = 0.03) but not the incidence of major complications, i.e., need of blood transfusions or urgent angiography (19). However, there are also studies that have not identified any utility of desmopressin prophylactic use in reducing minor or major bleeding complications after kidney biopsy (4, 11, 17).

Thus, prophylactic desmopressin seems indicated in patients at high risk of post-biopsy bleeding, namely those with eGFR <60 ml/min.

As desmopressin is a synthetic vasopressin receptor agonist which increases free water reabsorption in the collecting ducts, it can lead to iatrogenic hyponatremia if appropriate free water restriction is not achieved (10, 11). Most of studies evaluated the effect of desmopressin on bleeding complications and did not use post-biopsy serum sodium monitoring protocols (16). Accordingly, there is scarce data regarding the risk of hyponatremia after a prophylactic single-dose desmopressin prior to kidney biopsy (Table 2) (4, 23, 25, 26, 28–33).

Nonetheless, some studies have raised concerns about the risk of hyponatremia after desmopressin prophylactic use in this setting (11, 14, 17, 19).

Awareness of hyponatremia after post-biopsy bleeding prophylaxis with desmopressin increased after a case report. Desmopressin (12 μg, intravenously) was given to two patients with kidney graft dysfunction and low eGFR (34 and 22 ml/min), both with polyuria-driven high fluid intake. Profound (<125 mmol Na/l) and severe symptomatic (headache, vomiting, muscle cramps, seizures) hyponatremia was noted 1 day after biopsy in both, and was followed by severe neurologic sequalae in one case (14).

A retrospective study evaluated a cohort of 436 patients at high risk of bleeding (eGFR < 30 ml/min) who underwent native and allograft kidney biopsies, 226 receiving desmopressin prophylaxis (median dose 0.2 μg/kg, intravenously). The incidence of severe hyponatremia (mean 122 mmol/l) was 6.9%, significantly higher in those who received desmopressin prophylaxis (10.7 vs. 3.0%, *p* = 0.002) (11). The risk factors associated with the development of severe hyponatremia were severe renal impairment, lower pre-biopsy serum sodium levels and a higher dose of desmopressin (11). Thus, prophylaxis with desmopressin, although reducing the bleeding incidence in high-risk patients, increases the risk of hyponatremia.

Another retrospective study conducted by Ho et al. evaluated 195 patients who underwent allograft kidney biopsies, 98 with desmopressin prophylaxis (0.3 μg/kg, intravenously). Desmopressin did not reduce post-biopsy bleeding complications, but increased by three-fold the risk of hyponatremia. Severe hyponatremia (<125 mmol/l) developed in seven cases (7%) and was symptomatic in two (seizures in one). Hyponatremia was associated with pre-biopsy serum sodium level and fluid intake (17).

In two other retrospective studies, in patients at high risk of bleeding (eGFR <60 ml/min; serum creatinine >1.5 mg/dl), hyponatremia incidence (<130 mmol/l) was 10 and 9%, respectively, all events being asymptomatic (18, 19). In one of these studies (19), desmopressin given intranasally (150 μg) reduced by two-fold the risk of post-biopsy bleeding but a decrease in sodium level was noted in 94% of patients, with a mean decrease of 4.35 mmol/l. The predictors of hyponatremia were a higher eGFR, a lower pre-biopsy serum sodium and a high spot urine sodium (19).

Thus, data from these studies suggest that incidence of hyponatremia (<130 mmol/l) after desmopressin use for bleeding prophylaxis in high-risk patients (eGFR <60 ml/min) is around 10% but is seldom symptomatic (11, 17–19). The major predictors of vasopressin-associated hyponatremia are high hypotonic oral fluid intake (=imposed by pre-existent polyuria or by biopsy protocol), lower pre-biopsy serum sodium, higher urinary spot sodium and higher desmopressin dose (11, 17, 19). Only one study reported a dose-effect relation, while in the rest of the studies similar doses were used (0.3–0.4 μg/kg) (11). Route of administration could also have a role, as noted by Lim et al. (11). Manno et al. and Peters et al. used the subcutaneous route and, in both studies, desmopressin reduced the risk of post-biopsy bleeding (13, 28). As previously noted, the area under the curve of desmopressin plasma concentration is the highest after subcutaneous administration (20, 21). Intranasal administration seems equally effective (19) but data on the oral route, which was used in our case, are missing.

Our patient had two risk factors for bleeding after percutaneous kidney biopsy (34), old age (60 years) and a low estimated GFR (43 ml/min). Accordingly, as part of our department protocol, desmopressin 60 μg was administered orally 1 h before the procedure. To note, the dose is substantially lower than used in previous studies (0.3–0.4 μg/kg intravenously and 150 μg intranasally) and was administered by oral route. No bleeding of renal origin was noted. However, the post-biopsy outcome was complicated by acute profound hyponatremia (123 mmol/l) with severe neurological symptoms (headache, nausea, and recurrent vomiting), suggestive of cerebral edema (Figure 2). Subsequent to recurrent vomiting, he developed a Mallory-Weiss syndrome, followed by a life-threatening upper gastrointestinal bleeding. Thus, desmopressin prophylaxis could result not only in severe symptomatic acute hyponatremia, *but also in other, unexpected, non-neurological complications*. Therefore, we suggest that the assessment of serum sodium should be included in the pre- and post-biopsy protocol of patient monitoring in those that received desmopressin prophylaxis. However, as the supporting data are scarce, this algorithm should be evaluated in an adequate clinical trial.

## Conclusion

In conclusion, the risks associated with desmopressin use as bleeding prophylaxis after kidney biopsy should be acknowledged and, although symptomatic acute hyponatremia is rare, its consequences can be severe. Accordingly, we suggest that in such situation, serum sodium level assessment should be included in pre- and post-biopsy patient's monitoring protocol, and patients should be advised to limit their water intake after kidney biopsy.

## Data Availability Statement

The original contributions presented in the study are included in the article/supplementary material, further inquiries can be directed to the corresponding author/s.

## Ethics Statement

Written informed consent was obtained from the individual(s) for the publication of any potentially identifiable images or data included in this article.

## Author Contributions

GI, BO, and AV: conceptualization and writing—review and editing. BO and AV: data curation and writing—original draft preparation. GI, AD, BC, and NC: patient management. GI: supervision. All authors have read and agreed to the published version of the manuscript.

## Conflict of Interest

The authors declare that the research was conducted in the absence of any commercial or financial relationships that could be construed as a potential conflict of interest.
